# Apixaban Concentrations and Effects on Coagulation in Patients With Nephrotic Syndrome

**DOI:** 10.1016/j.xkme.2025.101136

**Published:** 2025-10-10

**Authors:** Sarah Kelddal, Erik L. Grove, Camilla L. Duus, Louis B. Nygaard, Tilde Kristensen, Frank H. Mose, Jon W. Gregersen, Anne-Mette Hvas, Henrik Birn

**Affiliations:** 1 Department of Renal Medicine, Aarhus University Hospital, Aarhus, Denmark; 2 Department of Biomedicine, Aarhus University Health, Aarhus, Denmark; 3 Department of Cardiology, Aarhus University Hospital, Aarhus, Denmark; 4 Department of Clinical Medicine, Faculty of Health, Aarhus University, Aarhus, Denmark; 5 University Clinic in Nephrology and Hypertension, Department of Internal Medicine, Goedstrup Hospital, Goedstrup, Denmark; 6 Department of Nephrology, Aalborg University Hospital, Aalborg, Denmark; 7 Medical Diagnostics Center, Viborg Regional Hospital, Viborg, Denmark; 8 Faculty of Health, Aarhus University, Aarhus, Denmark

**Keywords:** Nephrotic syndrome, thromboembolism, thrombin generation, apixaban, low molecular weight heparin, direct oral anticoagulants

## Abstract

**Rationale & Objective:**

Venous thromboembolism is a serious complication of nephrotic syndrome (NS). Guidelines recommend prophylactic anticoagulation with warfarin or low molecular weight heparin. Although widely used for other conditions, data on direct oral anticoagulants in NS are limited. We explored the potential of standard-dose apixaban by assessing its steady-state concentrations and anticoagulant effects in patients with NS.

**Study Design:**

An open-label, single-arm, controlled interventional clinical trial.

**Setting & Participants:**

The study included adult patients with NS (plasma albumin levels < 25 g/L and urine albumin-creatinine ratio > 2,200 mg/g) with a primary glomerular disease compared with healthy individuals.

**Interventions:**

Patients with NS received weight-adjusted dalteparin for at least 4 days, followed by a washout period of ≥ 24 hours, before starting apixaban 5 mg twice daily for a minimum of 4 days.

**Outcomes:**

The primary outcome was the steady-state plasma concentration of apixaban. Secondary outcomes included thrombin generation measured at baseline, during dalteparin steady-state, and at 2.5, 8, and 24 hours after the first apixaban dose, as well as at steady state.

**Results:**

Mean steady-state plasma apixaban level was significantly lower in patients with NS (n=11) than in healthy individuals (n=10) (35 μg/L, 95% CI, 28-43 vs 51 μg/L, 95% CI, 39-64; *P* = 0.02). Despite this, mean endogenous thrombin potential was comparable at steady-state (1,096 nM/min, 95% CI, 868-1,355 vs 910 nM/min, 95% CI, 713-1,107; *P* = 0.19). In patients with NS, apixaban reduced in vivo thrombin generation markers (prothrombin fragment 1+2 and thrombin-antithrombin complex) more effectively than dalteparin.

**Limitations:**

The small sample size and short study duration may limit the generalizability of the findings.

**Conclusions:**

Patients with NS demonstrated lower plasma apixaban concentrations but maintained comparable anticoagulant effects compared to healthy individuals. Apixaban showed greater suppression of in vivo thrombin generation than dalteparin. This supports apixaban as a viable alternative for thromboprophylaxis in NS.

**Trial Registration:**

ClinicalTrials.gov: NCT 04850378; EudraCT: 2019-001212-29.

## Plain Language Summary

Nephrotic syndrome (NS) is associated with a high risk of venous thromboembolism. Although current guidelines recommend prophylactic treatment with low molecular weight heparin or warfarin, these options have limitations. We investigated apixaban, a direct oral anticoagulant, as a potential alternative. In this study, patients with NS received apixaban, and drug levels as well as coagulation markers were compared with healthy individuals. Although plasma concentrations of apixaban were lower in patients with NS, anticoagulant effects were maintained compared with healthy individuals. These findings suggest that apixaban may be a feasible option for thromboprophylaxis in NS, but larger studies are needed to assess long-term safety and efficacy.

## Introduction

Venous thromboembolic events (VTEs) are serious complications of nephrotic syndrome (NS), contributing to increased morbidity and mortality.^1^^,^^2^ NS is known to be associated with altered coagulation, characterized by a shift toward a prothrombotic state, evidenced by increased thrombin generation.^3^^,^^4^ Despite significant focus on VTE risk and recommendations for prophylactic anticoagulant treatment, the overall incidence of VTE remains considerable and as high as 25%.^2^^,^^5^, ^6^, ^7^ International guidelines recommend low molecular weight heparin (LMWH) or warfarin for patients with NS who are considered at the highest risk of VTE.^8^ However, there are no randomized studies available, and only a few small retrospective studies have addressed the safety and efficacy of LMWH^9^, ^10^, ^11^ or warfarin.^12^^,^^13^ The findings suggest a protective effect of prophylactic anticoagulant treatment in patients with NS but with a significant risk of bleeding complications.^14^ Importantly, there are several challenges associated with the use of LMWH or warfarin. LMWH requires daily subcutaneous injections, which can be cumbersome and may cause discomfort for the patients, although warfarin has a narrow therapeutic range and numerous potential drug interactions, making its dosing difficult to manage and requiring frequent monitoring.^15^

Only a limited number of studies have explored the pharmacokinetic profiles of LMWH in patients with NS. LMWH acts by forming a complex with antithrombin, inhibiting activated coagulation factor Xa, thereby disrupting the clotting cascade and preventing thrombin generation.^12^ The available studies are small and report mixed results. Although a study showed no significant difference in anti-FXa between patients with NS (n=42) and healthy individuals,^16^ another study suggested reduced efficacy in patients with severe NS (n=6).^12^

Direct oral anticoagulants (DOACs) are a newer class of anticoagulants that have been associated with improved safety profiles in other populations, including fewer serious bleeding events and reduced mortality rates.^17^, ^18^, ^19^ DOACs inhibit thrombin or activated factor Xa, thereby disrupting coagulation and suppressing the terminal phase of the coagulation cascade.^15^ Because DOACs are highly protein-bound (up to 95%),^20^^,^^21^ their use in NS is limited by concerns related to hypoalbuminemia altering their pharmacokinetic profiles and therapeutic efficacy. Low plasma albumin levels may potentially cause reduced drug concentrations, possibly either lowering efficacy or increasing the unbound drug fraction, raising the risk of bleeding complications.^22^ There is limited pharmacokinetic and clinical data on the use of DOACs in patients with NS. Pharmacologic studies^23^^,^^24^ suggested that the hypoalbuminemia in NS significantly impacts the pharmacokinetics of DOACs, resulting in lower than expected peak concentrations and suboptimal plasma levels at specific time points, potentially affecting therapeutic efficacy. Current evidence regarding the efficacy and safety of DOACs in patients with NS is limited. Preliminary findings from small studies suggest that DOACs may provide VTE protection with an acceptable safety profile.^20^^,^^24^, ^25^, ^26^

To further explore the potential of DOACs for prevention and treatment of VTE in NS, this study examined apixaban pharmacokinetic profiles and its effects on key coagulation regulators in patients with NS because of primary glomerular diseases and compared both with the effects of LMWH and to healthy individuals.

## Methods

### Design and Ethical Considerations

We conducted an open-label, single-arm, controlled, interventional clinical trial to investigate the effects of dalteparin and apixaban in patients with NS and to compare the pharmacokinetic profiles of the latter with healthy individuals. The study was approved by the Danish Medicines Agency (reference no. 2020061178) and the Danish Research Ethics Committees (reference no. 1-10-72-158-20) and registered with ClinicalTrials.gov (identifier: NCT04850378) and EudraCT (identifier: 2019-001212-29). All study procedures adhered to the principles of the Declaration of Helsinki. Written informed consent was obtained from each participant before any trial-related procedures. The study was monitored using the Good Clinical Practice unit at Aarhus University and Aalborg University in Denmark.

### Study Population

Patients with a first episode or a relapse of NS were recruited during hospitalization or visits to the renal outpatient clinic at 4 nephrology centers in Denmark. The inclusion criteria were as follows: (1) plasma albumin levels < 25 g/L; (2) urine albumin-creatinine ratio > 2,200 mg/g; (3) age ≥18 years; and (4) biopsy-verified diagnosis of minimal change disease, focal segmental glomerulosclerosis, or membranous nephropathy. Patients with diabetes were allowed if the condition was well managed with a hemoglobin A1c level < 65 mmol/mol. Exclusion criteria were as follows: (1) estimated glomerular filtration rate (eGFR) < 30 mL/min/1.73 m^2^; (2) anticoagulant treatment for other conditions; (3) known coagulation disorder; (4) VTE during the last 3 months; (5) contraindication to dalteparin and apixaban; and (6) clinical infection, malignancy, or pregnancy. Patients with NS were compared with healthy individuals fulfilling the same inclusion and exclusion criteria, but with normal plasma albumin levels, no proteinuria, and no evidence of kidney disease.

### Data Collection

Information on age, gender, body mass index, smoking habits, alcohol, comorbid conditions including heart failure, liver failure, and any history of arterial or VTEs or bleeding disorders, current medication, as well as kidney biopsy findings were obtained at baseline.

### Interventions and Study Protocol

**Figure 1:**
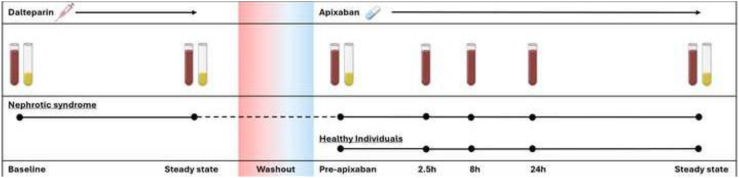
Study design and timing of blood and urine output samplings. Study design, including treatment phases, washout period, and sampling time points for both blood and urine output. Healthy individuals underwent baseline blood sampling before apixaban administration. Urine output samples included a spot urine output collection at baseline and a 24-hour urine output collection at the end of the study.

Patients with NS were first administered thromboprophylaxis with dalteparin, followed by apixaban as part of the study (Fig 1).

Following the collection of baseline blood and urine output samples, all patients with NS were prescribed weight-adjusted, subcutaneous dalteparin administered once daily using the following dosing scheme: ≤56 kg = 10,000 IU, 57-68 kg = 12,500 IU, 69-82 kg = 15,000 IU, and ≥83 kg = 18,000 IU. Dalteparin was administered for 4-7 days before collecting blood and 24-hour urine output samples. An additional blood sample was obtained exactly 4 hours after the last dalteparin dose to assess peak anti-Xa activity. Following a washout period of at least 24 hours, patients returned for new blood and urine output samples. Beginning at this visit, patients were administered 5 mg of oral apixaban, and blood samples were drawn 2.5 and 8 hours after dosing. The patients were prescribed apixaban at 5 mg twice daily and instructed to maintain precisely 12 hours between doses. Blood samples were collected after 24 hours and after 4-7 days of apixaban treatment.

Following baseline blood and urine output samples, healthy individuals were prescribed apixaban in the same manner as patients with NS, with blood samples taken at the same time points (Fig 1).

### Steady State Definition

Steady-state concentrations of apixaban have been reported to be achieved within 2-3 days of stable dosing.^21^ To ensure that a steady-state was attained in all participants, we included a minimum of 4 days from treatment initiation before collecting blood samples for steady-state analysis.

### Outcome Measurements

The primary outcome was the steady-state plasma concentration of apixaban in patients with NS compared to healthy individuals. Secondary outcomes were the effect of apixaban on thrombin generation markers, including endogenous thrombin potential (ETP), prothrombin fragment 1+2 (F1+F2), and thrombin-antithrombin complex (TAT), compared with healthy individuals. We compared the effect of apixaban and dalteparin on thrombin generation markers, assessing if comparable anticoagulant effects were achieved in patients with NS.

### Plasma Apixaban

Plasma apixaban concentrations were measured at 2,5, 8, and 24 hours after the first dose and at steady state (after ≥4 days of twice-daily dosing); the steady-state sample was collected immediately before the morning dose, representing a trough level (Fig 1).

### Thrombin Generation Markers

Thrombin generation was evaluated using both in vivo and ex vivo markers as described by Larsen et al.^27^ In vivo markers, including F1+F2 and TAT, indicate thrombin generation processes occurring within the body. In contrast, ex vivo markers such as ETP are derived from plasma-based assays to measure thrombin generation potential outside the physiologic system.

Thrombin generation markers were measured at baseline, after dalteparin steady state (days 4-7), at 2, 5, 8, and 24 hours after the first apixaban dose, and at apixaban steady state.

### Laboratory Analysis

Blood samples for plasma apixaban concentration, ETP, F1+F2, TAT, and 50% clot lysis time were drawn into 3.2% sodium citrate tubes. Samples were subjected to centrifugation at 3,000 g for 25 minutes at 20 °C to obtain platelet-poor plasma. The separated plasma was stored at −80 °C until analyzed. For ETP measurements, blood samples underwent an additional centrifugation at 2.500 g for 15 minutes at 20 °C before storage at −80 °C.

Plasma apixaban concentrations were quantified using Chromogenic anti-Xa assay specific for apixaban (STA-Liquid Anti-Xa Assay, CS 5100i; Sysmex). Samples were stored for a median of 70 days interquartile range (IQR, 50-195 days) before analysis.

F1+F2 concentration levels were measured using the Enzygnost F1+F2 (Monoclonal) enzyme-linked immunosorbent assay (Siemens Healthcare GmbH). TAT levels were quantified by the Enzygnost TAT essay (Siemens Healthcare Diagnostics).^28^ Results with a coefficient of variation exceeding 10% were reanalyzed, and TAT concentrations below the detection limit of 2 μg/L were assigned a value of 1 μg/L for analysis purposes. ETP was measured in platelet-poor plasma using calibrated automated thrombograms (Thrombinscope),^28^ using the area under the curve values (nM × min) as the final result.

Standard laboratory measurements included anti-Xa levels analyzed using the STA-Liquid Anti-Xa Assay (CS 5100i; Sysmex), plasma albumin (bromcresol purple method), creatinine concentrations determined using the Atellica CH analyzer (Siemens Healthcare GmbH), and complete blood counts conducted on an XN9000 analyzer (Sysmex). All routine analyses were performed by the Department of Clinical Biochemistry, Aarhus University Hospital, Denmark, in accordance with ISO15189-accredited routine protocols.

At baseline and at steady state, blood samples were also collected for hematology (hemoglobin levels, leukocyte and platelet counts), and coagulation markers (activated partial thromboplastin time, fibrinogen, and thrombin time), D-dimer, antithrombin, as well as plasma albumin and creatinine levels. Urine output samples included a spot urine for the urine albumin-creatinine ratio at baseline and 24-hour collections after each treatment period. Further blood samples were collected for coagulation parameters, thrombin generation markers, and plasma apixaban concentrations at 2.5, 8, and 24 hours after the first apixaban dose.

### Statistics

Baseline demographic characteristics are summarized using descriptive statistics. Categorical variables are reported as counts and percentages. Continuous variables were assessed for normality using QQ-plots. Normally distributed variables are reported as mean ± standard deviation, while non-normally distributed variables are reported as median with interquartile ranges (IQR; lower and upper quartiles). Mixed regression analysis was performed to evaluate plasma apixaban concentrations and thrombin generation assays across various timepoints, with results presented using line plots with mean and 95% confidence intervals (CIs). The steady-state concentration of apixaban and the effect of apixaban and dalteparin on thrombin generation were compared using an unpaired *t* test.

Linear regression analysis was performed to assess whether eGFR influenced plasma apixaban concentrations, with eGFR as the independent variable and apixaban concentration as the dependent variable.

All statistical analyses were conducted using Stata software version 17.0 (StataCorp). Figures were generated using GraphPad Prism version 10.3.1 (GraphPad Software).

## Results

### Demographics and Baseline Characteristics

A total of 11 patients with NS and 10 healthy individuals were included in the study. Patients with NS were older (mean age 51 ± 19 years), included a higher proportion of men (64% vs 40%), and had a greater prevalence of hypertension and heart failure than the healthy individuals (Table 1). Furthermore, patients with NS had a lower mean eGFR (81 ± 27 mL/min/1.73 m^2^ vs 107 ± 14 mL/min/1.73 m^2^), lower mean plasma albumin concentrations (19 ± 3 g/L vs 39 ± 1 g/L, *P* < 0.001), and higher median urine albumin-creatinine ratio (5,029 mg/g; IQR, 3,919-8,115 mg/g vs 6.5 mg/g; IQR, 2-11 mg/g; *P* < 0.001) than healthy individuals. The glomerular diagnoses among patients with NS included membranous nephropathy (n=5), minimal change disease (n=4), and focal segmental glomerulosclerosis (n=2). No differences in blood counts, natural anticoagulants, or hemostatic markers were observed between groups.

**Table 1:** Demographic and baseline characteristics for patients with nephrotic syndrome (NS) and healthy individuals

Parameter	NS (n=11)	Healthy individuals (n=10)
Male, n (%)	7 (64)	4 (40)
Age (y), mean ± SD	51 ± 19	36 ± 19
BMI (kg/m^2^), mean ± SD	28.5 ± 4.4	26.8 ± 6.4
Ever smoked, n (%)	5 (45)	2 (20)
Comorbid conditions, n (%)		
Hypertension	5 (45)	0 (0)
Heart failure	1 (9)	0 (0)
Liver disease	0 (0)	0 (0)
Diagnosis n (%)		
Membranous nephropathy	5 (45)	-
Minimal change disease	4 (36)	-
FSGS	2 (18)	-
Kidney function, n (%)		
eGFR, (mL/min/1.73 m^2^)		
eGFR > 90	4 (36)	8 (80)
eGFR 60-90	5 (45)	2 (20)
eGFR 30-59	2 (18)	0 (0)
Plasma albumin levels (g/L), mean ± SD	19 ± 2.6	39 ± 2.6
Median uACR (mg/g) (IQR)	5,029 (3,919-8,115)	6.5 (2-11)
Inflammatory and metabolic marker levels		
C-reactive protein levels (mg/L) (reference rnage, < 8 mg/L)	4.63 ± 2.0	-
Glycated hemoglobin levels (mmol/mol) (reference range, < 48 mmol/mol)	37 ± 5.7	34 ± 4.8
Blood counts		
Hemoglobin levels (mmol/L) (reference range, 7.3-10.5 mmol/L)	8.8 ± 0.9	8.5 ± 1.10
White blood cell counts (10^9^/L) (reference range, 3.5-10.010^9^/L)	8.0 ± 1.9	5.9 ± 1.0
Platelet counts (10^9^/L) (reference range, 145-400 10^9^/L)	314 ± 101	276 ± 86
Natural anticoagulant		
Antithrombin functional (10^3^ IU/L) (reference range, 0.80-1.20 10^3^ IU/L)]	0.87 ± 0.17	1.02 ± 0.15
Hemostatic marker levels at baseline		
Thrombin time (s) (reference range, <21s)	18 ± 1.5	17 ± 1.0
aPTT (s) (Reference range, 22-29 s)	24 ± 2.0	23 ± 1.8
Thrombin generation marker levels		
Median F1+F2 levels (pmol) (IQR)	383 (305-545)	170 (152-216)
TAT levels (μmol)	3.3 ± 1.0	3.1 ± 1.9
Median ETP levels (nmol × min) (IQR)	1,337 (1,132-1,634)	1,113 (985-1,608)

*Note:* Demographics and baseline characteristics of patients with NS and healthy individuals. Continuous variables are presented as mean ± SD unless explicitly stated as median with IQR. Categorical variables as counts and percentages. The table includes data on demographic characteristics, kidney function, blood counts, and baseline hemostatic marker levels for both groups.

Abbreviations: aPTT, activated partial thromboplastin time; BMI, body mass index; eGFR, estimated glomerular filtration rate; ETP, endogenous thrombin potential; F1+F2, prothrombin fragment 1+2; FSGS, focal segmental glomerulosclerosis; IQR, interquartile range; SD, standard deviation; TAT, thrombin-antithrombin complex; uACR, urine albumin-creatinine ratio.

### Plasma Apixaban Levels

**Figure 2:**
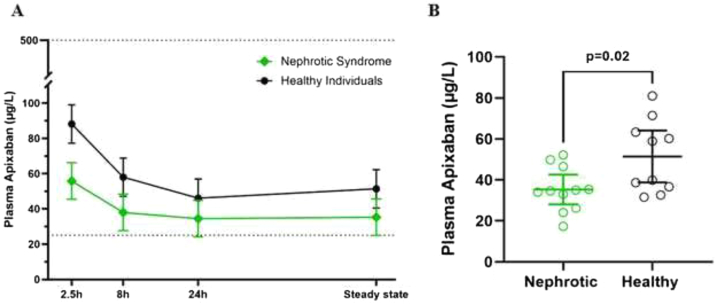
Plasma concentrations of apixaban in patients with nephrotic syndrome (NS) and healthy individuals over time. (A) Plasma apixaban concentrations at different time points in patients with NS (green) and healthy controls (black). Data are means with 95% confidence intervals. (B) Dot plot comparing plasma apixaban concentrations measured at steady state between a patient with NS (green) and healthy individuals (black).

Plasma apixaban concentrations were lower in patients with NS than in healthy individuals across all time points, as shown in Fig 2A. Mixed regression analysis showed a significant difference in the overall trajectories between the 2 groups (*P* = 0.02). At steady state, mean plasma apixaban concentration was significantly lower in patients with NS than in healthy individuals (35 μg/L, 95% CI, 28-43 vs 51 μg/L, 95% CI, 39-64; *P* = 0.02, unpaired *t* test, Fig 2B).

There was no significant association between eGFR and plasma apixaban concentrations among patients with NS (β of −0.072, 95% CI, −0.36 to 0.21, *P* = 0.58).

### Thrombin Generation Markers

**Figure 3:**
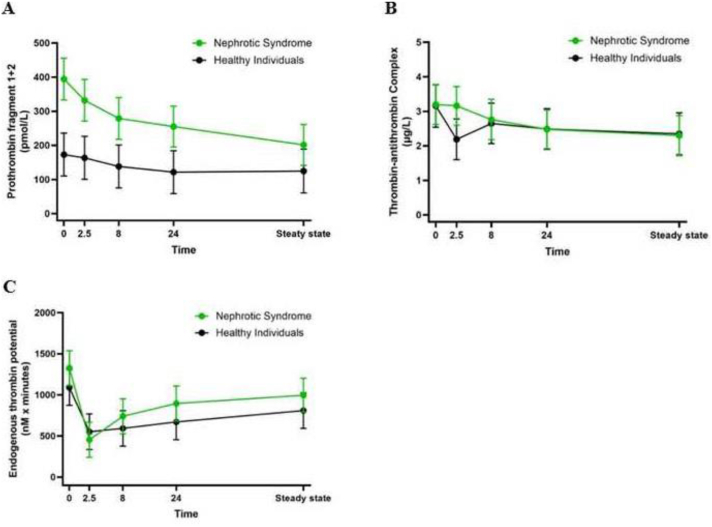
In vivo and ex vivo thrombin generation at baseline and following apixaban therapy in patients with nephrotic syndrome (NS) and healthy individuals. Line plots of ex vivo thrombin generation (endogenous thrombin potential) (A), in vivo thrombin generation markers (prothrombin fragment 1+2) (B), and thrombin-antithrombin complex levels (C) at baseline and following apixaban administration. Data for patients with NS (green) and healthy individuals (black) represent means with 95% confidence intervals across all time points.

Patients with NS exhibited markedly higher baseline F1+F2 levels than healthy individuals; however, levels decreased significantly over time in both patients with NS and healthy individuals following apixaban administration (Fig 3A). At steady-state apixaban levels, mean F1+F2 levels remained significantly higher in patients with NS than in healthy individuals (223 pmol/L, 95% CI, 175-268 vs 145 pmol/L, 95% CI, 98-191; *P* = 0.02). There was no significant difference in TAT levels between patients with NS and healthy individuals, neither at baseline nor after apixaban treatment, although it appeared lower in healthy individuals at 2.5 hours (Fig 3B). At steady state, mean TAT concentrations were comparable between groups (2.2 μmol/L, 95% CI, 1.5-2.9 in patients with NS vs 2.2 μmol/L, 95% CI, 1.5-3.0 in healthy individuals; *P* = 0.95). Moreover, no significant differences were observed in ETP levels between patients with NS and healthy individuals (Fig 3C). After apixaban treatment, mean steady-state ETP levels were comparable between the groups (1,096 mmol × min, 95% CI, 868-1,324 in patients with NS vs 910 mmol × min, 95% CI, 713-1,107 in healthy individuals; *P* = 0.18).

### Thrombin Generation Markers During Dalteparin and Apixaban Treatment

Among the 11 patients with NS treated with apixaban, 9 received dalteparin before apixaban with a washout period between the 2 treatments. When measured after a minimum of 4 days after initiation of dalteparin treatment, both the mean trough and mean peak anti-Xa activities (measured 4 hours postinjection) were within therapeutic ranges. The mean peak anti-Xa activity was 1.02 IU/mL (range, 0.62-1.55), which is close to the recommended target of 0.5-1.0 IU/mL for once daily therapeutic dosing.

**Figure 4:**
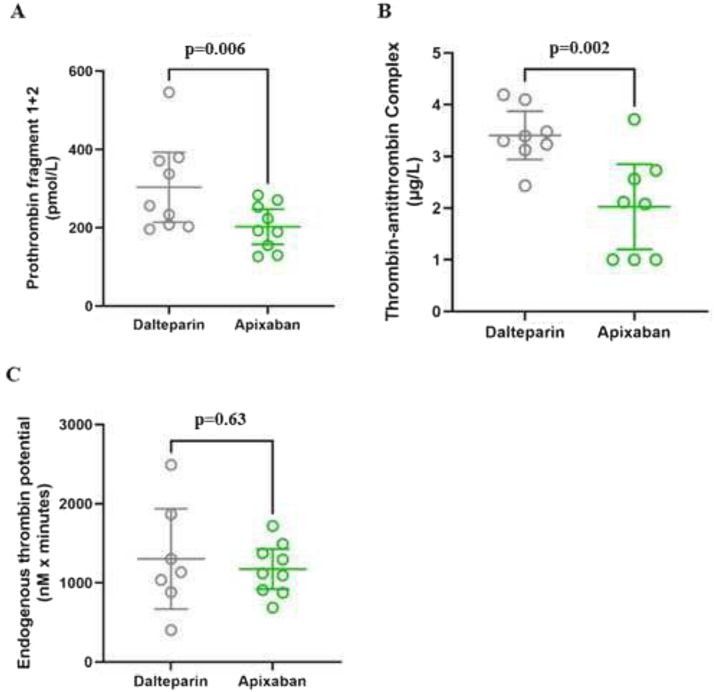
Steady-state thrombin generation markers in patients with nephrotic syndrome (NS) during treatment with dalteparin and apixaban. Thrombin generation markers in patients with NS at steady state following dalteparin (gray) and apixaban (green) treatment, including prothrombin fragment 1+2 (A), thrombin-antithrombin complex (B), and endogenous thrombin potential (C). Data are presented as dot plots, means, and 95% confidence intervals.

The mean of the in vivo thrombin generation marker F1+F2 was significantly lower during apixaban treatment than during dalteparin (203 μg/L, 95% CI, 157-248 vs 303 μg/L, 95% CI, 214-393; *P* = 0.002) (Fig 4A). Similarly, mean TAT levels were significantly lower during apixaban treatment than during dalteparin (2.0 μg/L, 95% CI, 1.2-2.8 vs 3.4 μg/L, 95% CI, 2.9-3.9; *P* = 0.006) (Fig. 4B). In contrast, no significant difference was observed in the mean ex vivo thrombin generation marker ETP, comparing apixaban treatment with dalteparin treatment (1,172 nmol × min, 95% CI, 1,141-1,915 vs 1,302 nmol × min, 95% CI, 668-1,935; *P* = 0.63, Fig 4C).

### Adverse Events During Apixaban Treatment

Adverse events were systematically assessed at steady state and again 7 days after treatment cessation. No VTE or major bleeding events were observed during the apixaban treatment period. Among patients with NS, 2 minor bleeding events were reported. One patient experienced known intermittent epistaxis, which also occurred during apixaban treatment, and another reported well-known intermittent hemorrhoidal bleeding, which similarly occurred under apixaban treatment. Additionally, 1 healthy individual reported slightly heavier menstrual bleeding while receiving apixaban. None of the bleeding episodes required medical intervention or adjustments to the anticoagulant medication dosage.

## Discussion

Following apixaban administration, patients with NS demonstrated significantly lower plasma apixaban concentrations than healthy individuals; however, thrombin generation markers reflecting the anticoagulant effectiveness were similar between the 2 groups. Furthermore, in vivo markers of thrombin generation, such as F1+F2, which were significantly elevated at baseline in patients with NS, progressively decreased during apixaban treatment, reaching levels comparable with healthy individuals at steady state. These findings suggest that apixaban effectively lowers thrombin generation in patients with NS, potentially lowering the risk of VTE.

Our findings are supported by 2 previous studies demonstrating lower plasma concentrations of apixaban and rivaroxaban in patients with NS compared with healthy individuals with normal plasma albumin levels.^23^^,^^24^ Notably, these studies highlighted the potential effect of hypoalbuminemia on the pharmacokinetic profiles of DOACs, consistent with the reduced apixaban concentration levels observed in our study. Furthermore, similar to our findings, no significant difference in ETP between patients with NS and healthy individuals was reported in a small trial (n=8) following a single dose of apixaban.^23^ This suggests that the anticoagulant effect, as measured by ETP, may not be impaired by the lower plasma apixaban concentration observed in patients with NS.

Dalteparin treatment is widely used and recommended for the prevention of VTE by international guidelines^8^; however, we observed no difference in ex vivo thrombin generation compared to apixaban, whereas in vivo thrombin generation markers, such as F1+F2 and TAT levels, were significantly lower during apixaban treatment. This difference likely reflects differences in their mechanisms of action. Apixaban, as a direct factor Xa inhibitor, effectively blocks thrombin generation during both the initiation and propagation phases of the coagulation cascade, independently of antithrombin.^21^ In contrast, dalteparin depends on antithrombin as a cofactor, and its efficacy may be reduced in nephrotic patients with hypoalbuminemia and low antithrombin levels.^29^ The lack of differences in ex vivo thrombin generation furthermore suggests that apixaban’s superior effect on in vivo thrombin generation may depend on dynamic processes, including interactions with endothelial cells and platelets, which are not captured in ex vivo systems. To our knowledge, this is the first study to directly compare thrombin generation markers in patients with NS during treatment with apixaban and dalteparin, respectively.

A key strength of our study is the prospective design with clearly defined inclusion criteria, ensuring a well-characterized study population. The systematic and time-defined sampling of blood and urine output further enhances the reliability and reproducibility of our findings. Moreover, the inclusion of patients with severe NS and a mean plasma albumin level of 19 g/L represents a high-risk population, who generally pose the greatest challenge in anticoagulant management, in part because of the altered pharmacokinetics of highly protein-bound drugs like apixaban. By focusing on steady-state apixaban concentrations, our study provides clinically relevant insights beyond those offered by single-dose studies. Additionally, the analyses of both in vivo and ex vivo thrombin generation markers enhance the robustness of our findings. On the other hand, limitations include the relatively small sample size, which reduces the power to detect minor differences in thrombin generation markers. Furthermore, the treatment duration of 4-7 days precludes assessment of long-term outcomes, such as VTE or bleeding complications. The study duration was chosen to allow for assessment of steady-state pharmacokinetics and anticoagulant effect, which is typically reached within 2-3 days of stable apixaban dosing.^21^ Although thrombin generation markers are valuable surrogate markers for anticoagulant efficacy in short-term studies, they cannot substitute for clinical outcomes in evaluating long-term safety and effectiveness. Thus, future studies with extended follow-up are warranted to assess the correlation between laboratory-based markers and clinical endpoints in this high-risk population. Finally, because our study focused exclusively on apixaban and patients with primary glomerular diseases, the generalizability to other DOACs and other causes of NS is uncertain. In addition, variability in the underlying glomerular diagnoses and severity of NS may have influenced pharmacokinetic and pharmacodynamic outcomes, but subgroup analyses were not feasible because of the limited sample size. Furthermore, the duration of hypoalbuminemia before inclusion was not systematically recorded, which may be a confounding factor influencing apixaban pharmacokinetic profiles.

In conclusion, patients with NS have significantly lower plasma apixaban concentrations than healthy individuals; however, apixaban effectively suppressed thrombin generation markers, achieving levels comparable with healthy individuals at steady state, and demonstrated greater suppression of in vivo thrombin generation markers compared with dalteparin. These findings support the use of apixaban in patients with NS. Future studies should evaluate the long-term safety and efficacy of treatment with apixaban to prevent VTE in this high-risk population.
